# Supplementary material for: Direct comparison of contralateral bias and face/scene selectivity in human occipitotemporal cortex
Source: Brain Struct Funct. 2021 Nov 2;227(4):1405–21. doi: 10.1007/s00429-021-02411-8 (PMC9046350; doi:10.1007/s00429-021-02411-8)
Supplement: Supplementary file 1 — Supplementary file1 (DOCX 17 KB) [file 429_2021_2411_MOESM1_ESM.docx]

## Supplementary Material

***Response magnitudes for all conditions***

Response magnitudes were submitted to a two-way repeated measures ANOVA with Position (Ipsilateral, Contralateral) and Category (non-preferred, preferred) as within-participant factors for each ROI separately. A significant Position by Category interaction was present in all ROIs (see breakdown below). We now include these statistics in the revised manuscript.

OFA: Both main effects of Position (F(17)=47.32, *p*=2.67-6) and Category (F(17)=47.92, *p*=2.46-6) were significant, as was the Position by Category interaction (F(17)=44.28, *p*=4.06-6).

FFA: Both main effects of Position (F(17)=67.56, *p*=2.51-7) and Category (F(17)=38.91, *p*=9.00-6) were significant, as was the Position by Category interaction (F(17)=15.07, *p*=1.19-3).

OPA: Both main effects of Position (F(17)=80.29, *p*=7.54-8) and Category (F(17)=58.25, *p*=6.88-7) were significant, as was the Position by Category interaction (F(17)=53.52, *p*=1.20-6).

PPA: Both main effects of Position (F(17)=47.52, *p*=2.60-6) and Category (F(17)=93.07, *p*=2.61-8.) were significant, as was the Position by Category interaction (F(17)=76.93, *p*=1.01-7).

#### Consistency of contralateral and category biases

***Session 1***

Only the main effect of Category (F(1, 17)=5.13, p=0.03) was significant, reflecting on average larger bias values in scene- over face-selective ROIs (p>0.05 for all other main effects). The Surface by Category (F(1, 17)=5.19, p=0.03) interaction was significant, again reflecting a larger difference in category bias between PPA and OPA. The Surface by Bias interaction (F(1, 17)=93.93, p=2.44-8) was also significant and reflects on average a greater contralateral bias laterally but a greater category bias ventrally (p>0.05, for all other interactions). A series of paired *t*-tests were performed comparing the contralateral versus category bias in each ROI separately (OFA: Contralateral v Category (t(17)=1.28, p=0.21), FFA: Contralateral v Category (t(17)=2.50, p=0.02), OPA: Contralateral v Category (t(17)=2.10, p=0.04), PPA: Contralateral v Category (t(17)=3.53, p=0.002) **(Figure 4A)**.

***Session 2***

Only the main effect of Bias (F(1, 17)=6.85, p=0.01) was significant, reflecting on average larger Category over Contralateral biases across ROIs (p>0.05, for all other main effects). The Surface by Category (F(1, 17)=4.48, p=0.04) interaction was significant, again reflecting a larger difference in category bias between PPA and OPA, but crucially so was the Surface by Bias interaction (F(1, 17)=137.11, p=2.10-9), (p>0.05, for all other interactions). Again, the Surface by Bias interaction is driven by a greater contralateral bias laterally, but a greater category bias ventrally (OFA: Contralateral v Category (t(17)=0.69, p=0.49), FFA: Contralateral v Category (t(17)=4.59, p=0.0002), OPA: Contralateral v Category (t(17)=2.197, p=0.008), PPA: Contralateral v Category (t(17)=4.28, p=0.0005) **(Figure 4B)**.

***Session 3***

Only the main effect of Category (F(1, 17)=4.76, p=0.04) was significant, reflecting on average larger bias values in scene- over face-selective ROIs (p>0.05) for all other main effects). Only the Surface by Bias (F(1, 17)=63.65, p=3.78-7) interaction was significant (p>0.05, for all other interactions). Again, the Surface by Bias interaction is driven by a greater contralateral bias laterally, but a greater category bias ventrally (OFA: Spatial v Category (t(17)=1.90, p=0.07), FFA: Spatial v Category (t(17)=3.90, p=0.001), OPA: Spatial v Category (t(17)=2.12, p=0.04), PPA: Spatial v Category (t(17)=2.88, p=0.01) **(Figure 4C)**.
